# Supplementary material for: Hybrid Models and Biological Model Reduction with PyDSTool
Source: PLoS Comput Biol. 2012 Aug 9;8(8):e1002628. doi: 10.1371/journal.pcbi.1002628 (PMC3415397; doi:10.1371/journal.pcbi.1002628)
Supplement: Text S4 — Complete source code for the PyDSTool package (version 0.88.120504). Includes API documentation and help files linking to web pages. This file is identical to the current public release on Sourceforge.net. (ZIP) [file pcbi.1002628.s004.zip › PyDSTool/html/PyDSTool.Toolbox.dataanalysis-module.html]

xml version="1.0" encoding="ascii"?


PyDSTool.Toolbox.dataanalysis


| Home | Trees | Indices | Help | PyDSTool |
| --- | --- | --- | --- | --- |

Package PyDSTool ::
Package Toolbox ::
Module dataanalysis

[hide private]

[frames] | no frames]

# Module dataanalysis

source code

Data analysis utilities

Robert Clewley, August 2006


|  |  |
| --- | --- |
| Classes | [hide private] |

data\_bins  
Class for data binning.


|  |  |
| --- | --- |
| Functions | [hide private] |

|  |  |
| --- | --- |
| doPCA(in\_pts, D, tol=0.8) | source code |

|  |  |
| --- | --- |
| get\_evec(p, dim) | source code |

|  |  |
| --- | --- |
| pca\_dim(pca, covering, data, refpt, tol=0.8) | source code |

|  |  |
| --- | --- |
| get\_residual(pcanode, pts) | source code |

|  |  |
| --- | --- |
| plot\_PCA\_residuals(data, D=None, newfig=True, marker=`'``o``'`, silent=False) | source code |

|  |  |
| --- | --- |
| plot\_PCA\_spectrum(data, D) | source code |

|  |  |
| --- | --- |
| log\_distances(m, sampleix=0, doplot=True, quiet=True, logv=None, plotstyle=None, return\_ixs=False)  Log distances (L2-norm) of data points from a reference point (sampleix). | source code |

|  |  |
| --- | --- |
| log\_distances\_with\_D(m, sampleix, logv=None, return\_ixs=False)  Log distances (L2-norm) of data points from a reference point (sampleix). | source code |

|  |  |
| --- | --- |
| get\_linear\_regression\_residual(pfit, x, y, weight=`'``'`, w=0)  Also see scipy.stat.linregress for a more sophisticated version | source code |

|  |  |
| --- | --- |
| fitline(x, y, lo=0, hi=1, doplot=True, quiet=True, linewidth=2)  fitline takes the position of low and high fit limits and returns the slope. | source code |

|  |  |
| --- | --- |
| find\_closest\_val(x, a, eps=1e-07, which\_norm=2)  Find one value in a closer than eps to x | source code |

|  |  |
| --- | --- |
| find\_nearby\_ball(data, refix, r, which\_norm=2, include\_ref=False)  Return indices of all points in data that are inside a ball of radius r from the reference point (not included). | source code |

|  |  |
| --- | --- |
| find\_nearby\_annulus(data, refix, rlo, rhi, which\_norm=2)  Return a list containing input data's indices of all neighbours of the reference point within the range of distances d such that dlo < d < dhi | source code |

|  |  |
| --- | --- |
| find\_central\_point(data, which\_norm=2)  Find the index of a point closest to the 'centre' of a data set. | source code |

|  |  |
| --- | --- |
| find\_recurrences(data, refix, r, times=None, ignore\_small=0, which\_norm=2)  Find recurrences of a trajectory in a ball of radius r centred at the reference point given by refix in data. | source code |

|  |  |
| --- | --- |
| find\_diameter(data, eps, which\_norm=2)  Find approximate diameter of data set, up to tolerance defined by eps > 0. | source code |

|  |  |
| --- | --- |
| dist\_between\_datasets(data1, data2, which\_norm=2) | source code |

|  |  |
| --- | --- |
| out\_of\_seq(a, inc\_only=False)  Determine if and where an integer array a is not increasing or decreasing in sequence by 1. | source code |

|  |  |
| --- | --- |
| whiten(data)  Subtract mean and divide by standard deviation in each column of data | source code |

|  |  |
| --- | --- |
| find\_knees(data, tol=1.0, inlog=False, verbose=False)  Find one or more 'knees' in data, according to high second derivatives. | source code |

|  |  |
| --- | --- |
| recurrence\_times(ts, partitions)  Internal function for use by find\_recurrences | source code |

|  |  |
| --- | --- |
| find\_from\_sorted(x, v, next\_largest=1, indices=None) | source code |

|  |  |
| --- | --- |
| colormap(mag, cmin, cmax)  Return a tuple of floats between 0 and 1 for the red, green and blue amplitudes. | source code |

|  |  |
| --- | --- |
| dump\_progress(x, tot, filename=`'``progress_info.dat``'`)  Send progress report to a file | source code |

|  |  |
| --- | --- |
| second\_diff(data, i)  Naive second difference formula at position i of data | source code |

|  |  |
| --- | --- |
| \_adjust\_ix(i, n)  Internal helper function | source code |


|  |  |
| --- | --- |
| Variables | [hide private] |

acosh

replaceSepStr

newton\_meth

float96

protected\_randomnames

ONES

typeCounter

specialfns

ensuredecimalconst

sign = `<ufunc 'sign'>`

toCircumflexSyntax

mathlookup

Continuous = `Continuous Domain`

toPowSyntax

rerr = `<PyDSTool.Redirector.Redirector object at 0x4d185b0>`

sym2name

sinh = `<ufunc 'sinh'>`

getlftrtD

doadd

less\_equal = `<ufunc 'less_equal'>`

replaceCallsWithDummies

randomlookup

cPickle

replaceSepQSpec

feval\_map\_const

protected\_auxnamesDB = `ModelSpec internal helper class: auxfnD...`

Inf = `inf`

specTypes = `('RHSfuncSpec', 'ImpFuncSpec', 'ExpFuncSpec')`

MReg

logical\_or = `<ufunc 'logical_or'>`

errorfields = `{0: ['t', 'error info'], 10: ['t', 'event list']}`

tan = `<ufunc 'tan'>`

math\_dir

atanh

name\_chars\_RE

temp\_macro\_names\_inv

arccos = `<ufunc 'arccos'>`

isVectorClause

POW\_STR

convert\_power\_reserved\_keywords

W\_NONTERMSTATEBD = `21`

predicate\_op

cosh = `<ufunc 'cosh'>`

dsInterface

inverseMathNameMap = `{'Random': 'Random', 'SystemRandom': 'Sys...`

degrees = `<ufunc 'degrees'>`

greater = `<ufunc 'greater'>`

E\_NONUNIQUETERM = `10`

radians = `<ufunc 'radians'>`

sin = `<ufunc 'sin'>`

symbolMapClass

token

API = `API_class()`

fmod = `<ufunc 'fmod'>`

ensureints

API\_class

funcnames

mathNameMap = `{'Abs': 'abs', 'Acos': 'acos', 'Asin': 'asin', '...`

alphabet\_chars\_RE

re

contained = `contained`

toDoubleStarSyntax

protected\_allnames = `['acos', 'asin', 'atan', 'atan2', 'ceil',...`

ensureparen

traceback

math\_globals

ZEROS

reserved\_keywords

arctan2 = `<ufunc 'arctan2'>`

ldexp = `<ufunc 'ldexp'>`

mapNames

trysimple

replaceSep

complex192

temp\_macro\_names

arctanh = `<ufunc 'arctanh'>`

string2ast

W\_UNCERTVAL = `0`

null\_predicate\_class

rout = `<PyDSTool.Redirector.Redirector object at 0x4d18cb0>`

dodiv

parserObject

simplify

minpack

feature\_node

isNameToken

W\_TERMSTATEBD = `11`

fit\_function

exp = `<ufunc 'exp'>`

DO\_POW

nameResolver = `ModelSpec internal helper class: nameResolver o...`

DomainType

findEndBrace

frexp = `<ufunc 'frexp'>`

simplify\_str

mapPowStr

ceil = `<ufunc 'ceil'>`

builtin\_auxnames

ensurebare

regObject

targetLangs = `['c', 'python', 'matlab']`

isfinite = `<ufunc 'isfinite'>`

allmathnames = `['acos', 'asin', 'atan', 'atan2', 'ceil', 'cos'...`

isnan = `<ufunc 'isnan'>`

strIfSeq

feval\_map\_symb

isIntegerToken

protected\_specialfns

cos = `<ufunc 'cos'>`

theGenSpecHelper = `GenSpecHelper()`

feature\_leaf

whoQ

dofun

replaceSepListInv

HAVE\_PSYCO = `True`

replaceSepInv

protected\_mathnames

utils\_info

pi = `3.14159265359`

asinh

arcsin = `<ufunc 'arcsin'>`

doneg

accrueCompTypes

count\_sep

uncertain = `uncertain`

protected\_macronames

tanh = `<ufunc 'tanh'>`

DO\_DEC

builtinlookup

getdim

fabs = `<ufunc 'fabs'>`

NaN = `nan`

sqrt = `<ufunc 'sqrt'>`

complex

ensureparen\_div

LargestInt32 = `2147483647`

allCompTypes

splitargs

splitastLR

dopower

TENS

processMultiDef

W\_NONTERMEVENT = `20`

joinStrs

qtypes

less = `<ufunc 'less'>`

W\_TERMEVENT = `10`

verstr\_parts = `['0', '7', '0']`

errmessages = `{0: 'Computation of trajectory failed', 10: 'Mor...`

process\_raw\_residual

W\_BISECTLIMIT = `12`

isNumericToken

isHierarchicalName

genDB = `Generator internal database class: []`

mod = `<ufunc 'remainder'>`

builtinFnSigInfo

warnfields = `{0: ['value', 'interval'], 10: ['t', 'event list'...`

Macheps

hypot = `<ufunc 'hypot'>`

ast2string

domul

remove\_indices\_from\_range

null\_predicate = `null_predicate_class(None)`

collect\_numbers

modf = `<ufunc 'modf'>`

power = `<ufunc 'power'>`

syms

Discrete = `Discrete Domain`

findNumTailPos

makeParList

num\_chars

readArgs

notcontained = `notcontained`

symbolMapDict = `{}`

resolveSpecTypeCombos

arcsinh = `<ufunc 'arcsinh'>`

joinAsStrs

proper\_match

log = `<ufunc 'log'>`

allmathnames\_symbolic = `['Acos', 'Asin', 'Atan', 'Atan2', 'Cei...`

log10 = `<ufunc 'log10'>`

ast2shortlist

modlookup

isToken

replaceSepList

dosub

arccosh = `<ufunc 'arccosh'>`

parser

E\_COMPUTFAIL = `0`

parseMatrixStrToDictStr

warnmessages = `{0: 'Uncertain value computed', 10: 'Terminal e...`

floor = `<ufunc 'floor'>`

arctan = `<ufunc 'arctan'>`

local\_fndef

protected\_scipynames

symbol

e = `2.71828182846`

NAMESEP

\_num\_types = `(<type 'float'>, <type 'int'>, <type 'numpy.float...`

\_int\_types = `(<type 'int'>, <type 'numpy.integer'>)`

\_seq\_types = `(<type 'list'>, <type 'tuple'>, <type 'numpy.ndar...`

PCANode = `None`

\_PCA\_utils = `['doPCA', 'get_evec', 'pca_dim', 'get_residual', ...`

\_metric\_utils = `['log_distances', 'log_distances_with_D']`

\_analysis\_utils = `['find_knees', 'whiten', 'find_closest_val',...`

\_fit\_utils = `['get_linear_regression_residual', 'fitline']`

dict\_tol = `array([ 1.00000000e-08])`

ALLOW\_THREADS = `1`

Abs = `Abs (ModelSpec wrapper)`

Acos = `Acos (ModelSpec wrapper)`

Asin = `Asin (ModelSpec wrapper)`

Atan = `Atan (ModelSpec wrapper)`

Atan2 = `Atan2 (ModelSpec wrapper)`

BUFSIZE = `10000`

Betavariate = `Betavariate (ModelSpec wrapper)`

CLIP = `0`

Ceil = `Ceil (ModelSpec wrapper)`

Choice = `Choice (ModelSpec wrapper)`

Cos = `Cos (ModelSpec wrapper)`

Cosh = `Cosh (ModelSpec wrapper)`

Degrees = `Degrees (ModelSpec wrapper)`

E = `QuantSpec e (ExpFuncSpec)`

ERR\_CALL = `3`

ERR\_DEFAULT = `0`

ERR\_DEFAULT2 = `2084`

ERR\_IGNORE = `0`

ERR\_LOG = `5`

ERR\_PRINT = `4`

ERR\_RAISE = `2`

ERR\_WARN = `1`

Exp = `Exp (ModelSpec wrapper)`

Expovariate = `Expovariate (ModelSpec wrapper)`

FLOATING\_POINT\_SUPPORT = `1`

FPE\_DIVIDEBYZERO = `1`

FPE\_INVALID = `8`

FPE\_OVERFLOW = `2`

FPE\_UNDERFLOW = `4`

Fabs = `Fabs (ModelSpec wrapper)`

False\_ = `False`

Floor = `Floor (ModelSpec wrapper)`

Fmod = `Fmod (ModelSpec wrapper)`

Frexp = `Frexp (ModelSpec wrapper)`

Gammavariate = `Gammavariate (ModelSpec wrapper)`

Gauss = `Gauss (ModelSpec wrapper)`

Getrandbits = `Getrandbits (ModelSpec wrapper)`

Getstate = `Getstate (ModelSpec wrapper)`

Hypot = `Hypot (ModelSpec wrapper)`

Infinity = `inf`

Jumpahead = `Jumpahead (ModelSpec wrapper)`

Ldexp = `Ldexp (ModelSpec wrapper)`

Log = `Log (ModelSpec wrapper)`

Log10 = `Log10 (ModelSpec wrapper)`

Lognormvariate = `Lognormvariate (ModelSpec wrapper)`

MAXDIMS = `32`

Max = `Max (ModelSpec wrapper)`

Min = `Min (ModelSpec wrapper)`

Modf = `Modf (ModelSpec wrapper)`

NAN = `nan`

NINF = `-inf`

NZERO = `-0.0`

Normalvariate = `Normalvariate (ModelSpec wrapper)`

PINF = `inf`

PZERO = `0.0`

Paretovariate = `Paretovariate (ModelSpec wrapper)`

Pi = `QuantSpec pi (ExpFuncSpec)`

Pow = `Pow (ModelSpec wrapper)`

RAISE = `2`

Radians = `Radians (ModelSpec wrapper)`

Randint = `Randint (ModelSpec wrapper)`

Random = `Random (ModelSpec wrapper)`

Randrange = `Randrange (ModelSpec wrapper)`

SHIFT\_DIVIDEBYZERO = `0`

SHIFT\_INVALID = `9`

SHIFT\_OVERFLOW = `3`

SHIFT\_UNDERFLOW = `6`

Sample = `Sample (ModelSpec wrapper)`

ScalarType = `(<type 'int'>, <type 'float'>, <type 'complex'>, ...`

Seed = `Seed (ModelSpec wrapper)`

Setstate = `Setstate (ModelSpec wrapper)`

Shuffle = `Shuffle (ModelSpec wrapper)`

Sin = `Sin (ModelSpec wrapper)`

Sinh = `Sinh (ModelSpec wrapper)`

Sqrt = `Sqrt (ModelSpec wrapper)`

Sum = `Sum (ModelSpec wrapper)`

Systemrandom = `Systemrandom (ModelSpec wrapper)`

Tan = `Tan (ModelSpec wrapper)`

Tanh = `Tanh (ModelSpec wrapper)`

True\_ = `True`

UFUNC\_BUFSIZE\_DEFAULT = `10000`

UFUNC\_PYVALS\_NAME = `'UFUNC_PYVALS'`

Uniform = `Uniform (ModelSpec wrapper)`

Vonmisesvariate = `Vonmisesvariate (ModelSpec wrapper)`

WRAP = `1`

Weibullvariate = `Weibullvariate (ModelSpec wrapper)`

Wichmannhill = `Wichmannhill (ModelSpec wrapper)`

absolute = `<ufunc 'absolute'>`

add = `<ufunc 'add'>`

bitwise\_and = `<ufunc 'bitwise_and'>`

bitwise\_not = `<ufunc 'invert'>`

bitwise\_or = `<ufunc 'bitwise_or'>`

bitwise\_xor = `<ufunc 'bitwise_xor'>`

c\_ = `<numpy.lib.index_tricks.CClass object at 0x1154330>`

cast = `{<type 'numpy.int64'>: <function <lambda> at 0x10a8bb0>...`

conj = `<ufunc 'conjugate'>`

conjugate = `<ufunc 'conjugate'>`

copysign = `<ufunc 'copysign'>`

deg2rad = `<ufunc 'deg2rad'>`

divide = `<ufunc 'divide'>`

equal = `<ufunc 'equal'>`

exp2 = `<ufunc 'exp2'>`

expm1 = `<ufunc 'expm1'>`

floor\_divide = `<ufunc 'floor_divide'>`

fmax = `<ufunc 'fmax'>`

fmin = `<ufunc 'fmin'>`

greater\_equal = `<ufunc 'greater_equal'>`

index\_exp = `<numpy.lib.index_tricks.IndexExpression object at ...`

inf = `inf`

infty = `inf`

invert = `<ufunc 'invert'>`

isinf = `<ufunc 'isinf'>`

left\_shift = `<ufunc 'left_shift'>`

little\_endian = `True`

log1p = `<ufunc 'log1p'>`

logaddexp = `<ufunc 'logaddexp'>`

logaddexp2 = `<ufunc 'logaddexp2'>`

logical\_and = `<ufunc 'logical_and'>`

logical\_not = `<ufunc 'logical_not'>`

logical\_xor = `<ufunc 'logical_xor'>`

maximum = `<ufunc 'maximum'>`

mgrid = `<numpy.lib.index_tricks.nd_grid object at 0x113d490>`

minimum = `<ufunc 'minimum'>`

multiply = `<ufunc 'multiply'>`

n = `9`

nan = `nan`

nbytes = `{<type 'numpy.int64'>: 8, <type 'numpy.int16'>: 2, <t...`

negative = `<ufunc 'negative'>`

newaxis = `None`

nextafter = `<ufunc 'nextafter'>`

not\_equal = `<ufunc 'not_equal'>`

ogrid = `<numpy.lib.index_tricks.nd_grid object at 0x113dbb0>`

ones\_like = `<ufunc 'ones_like'>`

r\_ = `<numpy.lib.index_tricks.RClass object at 0x11542f0>`

rad2deg = `<ufunc 'rad2deg'>`

reciprocal = `<ufunc 'reciprocal'>`

remainder = `<ufunc 'remainder'>`

right\_shift = `<ufunc 'right_shift'>`

rint = `<ufunc 'rint'>`

s\_ = `<numpy.lib.index_tricks.IndexExpression object at 0x11543f0>`

sctypeDict = `{0: <type 'numpy.bool_'>, 1: <type 'numpy.int8'>,...`

sctypeNA = `{'?': 'Bool', 'B': 'UInt8', 'Bool': <type 'numpy.bo...`

sctypes = `{'complex': [<type 'numpy.complex64'>, <type 'numpy....`

signbit = `<ufunc 'signbit'>`

spacing = `<ufunc 'spacing'>`

square = `<ufunc 'square'>`

subtract = `<ufunc 'subtract'>`

t = `'0'`

true\_divide = `<ufunc 'true_divide'>`

trunc = `<ufunc 'trunc'>`

typeDict = `{0: <type 'numpy.bool_'>, 1: <type 'numpy.int8'>, 2...`

typeNA = `{'?': 'Bool', 'B': 'UInt8', 'Bool': <type 'numpy.bool...`

typecodes = `{'All': '?bhilqpBHILQPfdgFDGSUVOMm', 'AllFloat': '...`


|  |  |
| --- | --- |
| Function Details | [hide private] |

|  |  |
| --- | --- |
| log\_distances(m, sampleix=0, doplot=True, quiet=True, logv=None, plotstyle=None, return\_ixs=False) | source code |

Log distances (L2-norm) of data points from a reference point
(sampleix).

For multiple calls to this function on the same data, it's faster to
pass logv precomputed.

return\_ixs=True also returns the point indices from the sort to be
returned in this argument in a third return value.

|  |  |
| --- | --- |
| log\_distances\_with\_D(m, sampleix, logv=None, return\_ixs=False) | source code |

Log distances (L2-norm) of data points from a reference point
(sampleix). Additionally, it returns a list of the inter-point distances,
relative to the reference point, and the inverse of this mapping.

For multiple calls to this function on the same data, it's faster to
pass logv precomputed.

return\_ixs=True returns the point indices from the sort in a fifth
return value. This identifies the points in the returned list of
inter-point distances and its inverse.

|  |  |
| --- | --- |
| fitline(x, y, lo=0, hi=1, doplot=True, quiet=True, linewidth=2) | source code |

fitline takes the position of low and high fit limits and returns the
slope. Integer indices in the x data set can be specified for the low and
high limits, otherwise use a fraction between 0 and 1.

In application to fractal dimension estimation, if x = log d (radius,
a.k.a. inter-point distance) and y = log v (index, a.k.a. estimate of
volume at a given radius) then the slope is the dimension estimate of the
data set.

Also see scipy.stat.linregress for a more sophisticated version

|  |  |
| --- | --- |
| find\_central\_point(data, which\_norm=2) | source code |

Find the index of a point closest to the 'centre' of a data set. The
centre is defined to be where the mean of the points' position vectors
relative to the centre is minimized in each direction.

|  |  |
| --- | --- |
| find\_recurrences(data, refix, r, times=None, ignore\_small=0, which\_norm=2) | source code |

```
Find recurrences of a trajectory in a ball of radius r centred at the
reference point given by refix in data.

If recurrence times are desired, pass an associated time array corresponding
to the points in the data.

If ignore_small > 0, recurrences of fewer than this number of data points
   will be treated as anomalous and ignored completely.

Returns a structure (class common.args) having attributes:
    partitions -- pairs of entry and exit indices for each recurrence in
            the ball
    ball_ixs -- all consecutive indices into data for each recurrence
    partitions_lengths -- number of indices in each partition
    rec_times -- times to next recurrence in the set from previous
```

|  |  |
| --- | --- |
| find\_diameter(data, eps, which\_norm=2) | source code |

Find approximate diameter of data set, up to tolerance defined by eps
> 0.

Data assumed to be a rank-2 array.

|  |  |
| --- | --- |
| find\_knees(data, tol=1.0, inlog=False, verbose=False) | source code |

Find one or more 'knees' in data, according to high second
derivatives.

inlog option finds knees in the logs of the data entries. tol=1. works
well if inlog=False tol=0.3 works well if inlog=True

|  |  |
| --- | --- |
| colormap(mag, cmin, cmax) | source code |

Return a tuple of floats between 0 and 1 for the red, green and blue
amplitudes. Function originally named floatRgb and written by Alexander
Pletzer, from the Python Cookbook.

  


|  |  |
| --- | --- |
| Variables Details | [hide private] |

### protected\_auxnamesDB

Value:
:   ```
    ModelSpec internal helper class: auxfnDBclass object
    ```

### inverseMathNameMap

Value:
:   ```
    {'Random': 'Random',
     'SystemRandom': 'Systemrandom',
     'WichmannHill': 'Wichmannhill',
     'abs': 'Abs',
     'acos': 'Acos',
     'asin': 'Asin',
     'atan': 'Atan',
     'atan2': 'Atan2',
    ...
    ```

### mathNameMap

Value:
:   ```
    {'Abs': 'abs',
     'Acos': 'acos',
     'Asin': 'asin',
     'Atan': 'atan',
     'Atan2': 'atan2',
     'Betavariate': 'betavariate',
     'Ceil': 'ceil',
     'Choice': 'choice',
    ...
    ```

### protected\_allnames

Value:
:   ```
    ['acos',
     'asin',
     'atan',
     'atan2',
     'ceil',
     'cos',
     'cosh',
     'degrees',
    ...
    ```

### nameResolver

Value:
:   ```
    ModelSpec internal helper class: nameResolver object
    ```

### allmathnames

Value:
:   ```
    ['acos',
     'asin',
     'atan',
     'atan2',
     'ceil',
     'cos',
     'cosh',
     'degrees',
    ...
    ```

### errmessages

Value:
:   ```
    {0: 'Computation of trajectory failed',
     10: 'More than one terminal event found'}
    ```

### warnfields

Value:
:   ```
    {0: ['value', 'interval'],
     10: ['t', 'event list'],
     11: ['t', 'var name', 'var value', '\n\tvalue interval'],
     12: ['t', 'event list'],
     20: ['t', 'event list'],
     21: ['t', 'var name', 'var value', '\n\tvalue interval']}
    ```

### allmathnames\_symbolic

Value:
:   ```
    ['Acos',
     'Asin',
     'Atan',
     'Atan2',
     'Ceil',
     'Cos',
     'Cosh',
     'Degrees',
    ...
    ```

### warnmessages

Value:
:   ```
    {0: 'Uncertain value computed',
     10: 'Terminal event(s) found',
     11: 'State variable reached bounds (terminal)',
     12: 'Bisection limit reached for event',
     20: 'Non-terminal event(s) found',
     21: 'State or input variable reached bounds (non-terminal)'}
    ```

### \_num\_types

Value:
:   ```
    (<type 'float'>,
     <type 'int'>,
     <type 'numpy.floating'>,
     <type 'numpy.integer'>)
    ```

### \_seq\_types

Value:
:   ```
    (<type 'list'>, <type 'tuple'>, <type 'numpy.ndarray'>)
    ```

### \_PCA\_utils

Value:
:   ```
    ['doPCA',
     'get_evec',
     'pca_dim',
     'get_residual',
     'plot_PCA_residuals',
     'plot_PCA_spectrum']
    ```

### \_analysis\_utils

Value:
:   ```
    ['find_knees',
     'whiten',
     'find_closest_val',
     'out_of_seq',
     'find_nearby_ball',
     'find_nearby_annulus',
     'data_bins',
     'find_central_point',
    ...
    ```

### ScalarType

Value:
:   ```
    (<type 'int'>,
     <type 'float'>,
     <type 'complex'>,
     <type 'long'>,
     <type 'bool'>,
     <type 'str'>,
     <type 'unicode'>,
     <type 'buffer'>,
    ...
    ```

### cast

Value:
:   ```
    {<type 'numpy.int64'>: <function <lambda> at 0x10a8bb0>, <type 'numpy.
    int16'>: <function <lambda> at 0x10a8bf0>, <type 'numpy.complex128'>: 
    <function <lambda> at 0x10a8c30>, <type 'numpy.uint64'>: <function <la
    mbda> at 0x10a8c70>, <type 'numpy.complex256'>: <function <lambda> at 
    0x10a8cf0>, <type 'numpy.float32'>: <function <lambda> at 0x10a8d30>, 
    <type 'numpy.bool_'>: <function <lambda> at 0x10a8cb0>, <type 'numpy.u
    int8'>: <function <lambda> at 0x10a8db0>, <type 'numpy.int32'>: <funct
    ion <lambda> at 0x10a8eb0>, <type 'numpy.int8'>: <function <lambda> at
    ...
    ```

### index\_exp

Value:
:   ```
    <numpy.lib.index_tricks.IndexExpression object at 0x11543b0>
    ```

### nbytes

Value:
:   ```
    {<type 'numpy.int64'>: 8, <type 'numpy.int16'>: 2, <type 'numpy.comple
    x128'>: 16, <type 'numpy.uint64'>: 8, <type 'numpy.bool_'>: 1, <type '
    numpy.complex256'>: 32, <type 'numpy.float32'>: 4, <type 'numpy.int8'>
    : 1, <type 'numpy.uint8'>: 1, <type 'numpy.uint16'>: 2, <type 'numpy.o
    bject_'>: 4, <type 'numpy.float64'>: 8, <type 'numpy.int32'>: 4, <type
     'numpy.string_'>: 0, <type 'numpy.void'>: 0, <type 'numpy.float128'>:
     16, <type 'numpy.int32'>: 4, <type 'numpy.uint32'>: 4, <type 'numpy.u
    nicode_'>: 0, <type 'numpy.complex64'>: 8, <type 'numpy.uint32'>: 4}
    ```

### sctypeDict

Value:
:   ```
    {0: <type 'numpy.bool_'>,
     1: <type 'numpy.int8'>,
     2: <type 'numpy.uint8'>,
     3: <type 'numpy.int16'>,
     4: <type 'numpy.uint16'>,
     5: <type 'numpy.int32'>,
     6: <type 'numpy.uint32'>,
     7: <type 'numpy.int32'>,
    ...
    ```

### sctypeNA

Value:
:   ```
    {'?': 'Bool',
     'B': 'UInt8',
     'Bool': <type 'numpy.bool_'>,
     'Complex128': <type 'numpy.complex256'>,
     'Complex32': <type 'numpy.complex64'>,
     'Complex64': <type 'numpy.complex128'>,
     'D': 'Complex64',
     'F': 'Complex32',
    ...
    ```

### sctypes

Value:
:   ```
    {'complex': [<type 'numpy.complex64'>,
                 <type 'numpy.complex128'>,
                 <type 'numpy.complex256'>],
     'float': [<type 'numpy.float32'>,
               <type 'numpy.float64'>,
               <type 'numpy.float128'>],
     'int': [<type 'numpy.int8'>,
             <type 'numpy.int16'>,
    ...
    ```

### typeDict

Value:
:   ```
    {0: <type 'numpy.bool_'>,
     1: <type 'numpy.int8'>,
     2: <type 'numpy.uint8'>,
     3: <type 'numpy.int16'>,
     4: <type 'numpy.uint16'>,
     5: <type 'numpy.int32'>,
     6: <type 'numpy.uint32'>,
     7: <type 'numpy.int32'>,
    ...
    ```

### typeNA

Value:
:   ```
    {'?': 'Bool',
     'B': 'UInt8',
     'Bool': <type 'numpy.bool_'>,
     'Complex128': <type 'numpy.complex256'>,
     'Complex32': <type 'numpy.complex64'>,
     'Complex64': <type 'numpy.complex128'>,
     'D': 'Complex64',
     'F': 'Complex32',
    ...
    ```

### typecodes

Value:
:   ```
    {'All': '?bhilqpBHILQPfdgFDGSUVOMm',
     'AllFloat': 'fdgFDG',
     'AllInteger': 'bBhHiIlLqQpP',
     'Character': 'c',
     'Complex': 'FDG',
     'Datetime': 'Mm',
     'Float': 'fdg',
     'Integer': 'bhilqp',
    ...
    ```

  


| Home | Trees | Indices | Help | PyDSTool |
| --- | --- | --- | --- | --- |

|  |  |
| --- | --- |
| Generated by Epydoc 3.0.1 on Fri May 4 15:24:04 2012 | http://epydoc.sourceforge.net |
